# Dissecting genetic risk factors in breast cancer

**DOI:** 10.18632/oncotarget.15089

**Published:** 2017-02-04

**Authors:** Carly M. Harro, Alvaro N.A. Monteiro

**Affiliations:** Department of Cell Biology, Microbiology, and Molecular Biology, Cancer Epidemiology Program, H. Lee Moffitt Cancer Center and Research Institute, College of Arts and Sciences, University of South Florida, Tampa, FL, USA

**Keywords:** genome-wide association study, breast cancer, chromatin features, susceptibility loci, enhancer

Breast cancer associated with germline pathogenic variants in *BRCA1* or *BRCA2* is recognized for its strong familial risk [1]. In addition to these *BRCA1* and *BRCA2* variants, other rare (allele frequency < 0.005) highly penetrant variants in *TP53*, *PTEN*, and *ATM* account for ~25% of breast cancer familial risk [2]. Other susceptibility genes have been identified (*PALB2*, *CHEK2, RECQL, NBN*) as well as a large number of low penetrance variants [[3] and references therein]. However, known variants account for only ~50% of breast cancer susceptibility illustrating the polygenic nature of breast cancer risk and indicating that variants contributing to breast cancer risk remain to be discovered [4].

Genome Wide Association Studies (GWAS) serve as a powerful approach for discovering common risk variants underlying disease etiology. Often these variants reside within the non-coding region of the genome making the determination of functional mechanisms driving susceptibility a challenging task [5]. Differences in expression due to common polymorphic alleles have been shown to affect a variety of phenotypes involved in human diseases as well as account for 30% of cis-regulation of genes [6]. In the recent study published by *Oncotarget*, Hamdi et al. exploited this concept to search for additional breast cancer susceptibility loci [8].

**Figure 1:**
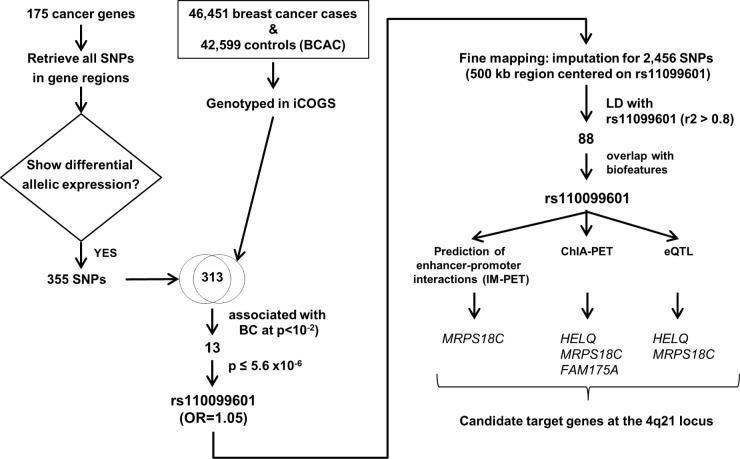
Outline of Hamdi et al. study identifying the 4q21 breast cancer susceptibility locus

In the study, Hamdi et al. evaluated 313 SNPs in 175 genes related to cancer for association with breast cancer risk in 46,451 breast cancer cancers and 42,599 controls of Caucasian ancestry participating in the Breast Cancer Association Consortium (BCAC) genotyped using the custom Illumina Infinium array iCOGS (Figure 1). First, the authors generated a list of genes implicated in cancer pathways using Kyoto Encyclopedia of Genes and Genomes (KEGG), a database which links genomic to functional information, and published data. Then, variants within these gene regions which had been previously reported for allelic expression *cis*-associations (*cis-*eQTL) were interrogated for association with breast cancer risk. Three SNPs, rs11099601, rs656040 and rs738200 were significantly associated (*P* < 10^-4^; correcting for multiple testing) with an increased risk of breast cancer. The most significant association with increased risk for both ER-positive and ER-negative breast cancer was rs110099601 (OR = 1.05, *P* = 5.6 × 10^-6^) at 4q21, which constitutes a novel breast cancer susceptibility locus [7].

To further explore molecular mechanisms driving susceptibility at the 4q21, 88 variants in strong linkage disequilibrium (r^2^ > 0.8) with rs11099601 were functionally annotated using ENCODE chromatin features such as overlapping with Histone epigenetic marks associated with promoters and enhancers or DNAse I hypersensitive sites (Figure 1). The variant rs11099601 displayed the strongest evidence of regulatory activity and was then assessed for interaction with potential target genes. Consistent with the chromatin features, multiple ChIA-PET (Chromatin Interaction Analysis coupled to paired-end tag sequencing) for RNA Polymerase II linking rs11099601 and the transcription start site of local genes *HELQ*, encoding a DNA dependent ATPase and DNA helicase, *MRPS18C*, which encodes the mitochondrial ribosomal protein S18C and *FAM175A* encoding Abraxas, a *BRCA1-*interacting protein [7] were identified. Interestingly, expression quantitative trait locus analysis (eQTL) was conducted in normal breast tissue and breast cancer datasets and showed an association of isoforms of *MRPS18C* and *HELQ* expression with rs11099601, albeit with a lack of consistency across different datasets. Taken together, these preliminary analyses suggest that breast cancer susceptibility is driven by changes in expression of multiple genes at the 4q21 locus, although further studies are needed to determine molecular mechanisms operating at the locus.

In summary, Hamdi et al. reported a novel association to breast cancer risk likely to be related to changes in expression of multiple genes. Importantly, although the approach used by Hamdi et al. may exclude the discovery of genes not previously implicated in cancer, it provides an example of an approach likely to identify variants undetectable under stringent GWAS multiple testing threshold (typically *p* ≤ 5 × 10^-8^), which may be particularly effective for tumor types with limited sample sets.
